# A post-tragedy psychiatric approach to violence prevention and community healing

**DOI:** 10.1192/j.eurpsy.2025.1525

**Published:** 2025-08-26

**Authors:** V. Vukovic, S. Nikolic Lalic, J. Mitić, M. Jakšić, A. Opankovic

**Affiliations:** 1 Clinical Hospital Centre “dr Dragiša Mišović”, Belgrade; 2 Special hospital for psychiatric disorders “dr Slavoljub Bakalović”, Vršac; 3 Institute for mental health; 4 Faculty of Medicine, Clinic for psychiatry, Clinical Centre of Serbia, Belgrade, Serbia

## Abstract

**Introduction:**

The world is increasingly burdened by complex, interwoven crises—economic, social, and political— and societies must examine and adapt their institutions to prevent and mitigate violence. Developing countries such as Serbia are transitioning from a history of conflict and face many difficulties in this regard. Lingering stigma around mental health, limited resources, and collective societal trauma complicate the task of responding to incidents of violence. One of these is the recent school shooting which has, on the one hand, highlighted deficiencies in early identification and intervention for at-risk adolescents, and has, on the other hand, pointed out the potential role of forensic psychiatry in transforming both prevention and post-crisis mediation.

**Objectives:**

The aim of this study will be to propose potential pathways to redefine the Serbian forensic psychiatric landscape, through delineating interdisciplinary interventions such as implementing community-based mental health education, expanding risk assessment protocols for youth, and providing prophylactic care for vulnerable populations.

**Methods:**

Drawing on comparative data from international practices, this paper explores several potential culturally adapted forensic models, focusing on early intervention, trauma-informed care, and interdisciplinary collaboration.

**Results:**

The dialogue between forensics and restorative justice could help define models for facilitating community healing, as well as enabling accountability and rehabilitation for patients. This paper proposes that changes in forensic psychiatry might facilitate the development of evidence-based frameworks directed at reducing future violent incidents, if applied in an communal, distributed model which includes social workers and other persons of interest. These interventions would be crucial in aligning Serbia’s forensic practices with current socio-political dynamics, potentially fostering a more effective and scientifically informed approach to justice and rehabilitation.

**Conclusions:**

Implementing a culturally adapted, interdisciplinary forensic psychiatric model in Serbia could provide a vital framework for violence prevention, community healing, and the integration of restorative justice practices.

**Disclosure of Interest:**

None Declared

